# Gender differences in HIV testing service visits and its related factors among adults: a cross-sectional study in Homa Bay, Kenya

**DOI:** 10.11604/pamj.2021.40.217.28331

**Published:** 2021-12-09

**Authors:** Kana Suzuki, Ryota Ochiai, Rose Okoyo Opiyo, Yuri Tokunaga, Yoko Imazu, Setsuko Watabe

**Affiliations:** 1 Department of Nursing, Graduate School of Medicine, Yokohama City University, Yokohama, Japan,; 2 School of Public Health, University of Nairobi, Nairobi, Kenya

**Keywords:** HIV testing services, testing behaviour, factors, gender differences, Homa Bay

## Abstract

**Introduction:**

at least 90% of people living with human immunodeficiency virus (HIV) were expected to know their HIV status by 2020. However, only 84% are aware of their status. This study determined the frequency of HIV testing services visits (HTS) and its related factors to HTS visits among adults in Homa Bay County, Kenya.

**Methods:**

this was a cross-sectional study. Quantitative and qualitative data were collected. A backward stepwise logistic regression analysis was conducted for quantitative data by gender. Qualitative data were thematically categorised into factors of HTS visits by gender.

**Results:**

a total of 645 adults participated in quantitative survey and 17 in qualitative survey. There were no gender differences in the frequency of HTS visits (males=56.3%; females= 58.7%, P=0.785). The frequency of visits was however significantly different between the rural-based (Rachuonyo North=87.5%; Ndhiwa=58.7%) and urban-based (Homa Bay Town=36.8%) facilities at P<0.001. In males, HTS visits were positively associated with ´being in Protestant church´, ´partner´s attitude´, and ´being accompanied by a friend to HTS´. ´Distance to HTS´ was negatively associated with HTS visits in males. For females, 'sexual intercourse in the past 2-5 months´ was positively associated with HTS visits. ´Being in a polygamous marriage´, ´not married´, ´community HIV testing´, and ´affordability of transport cost to HTS centre´ were negatively associated with HTS visits.

**Conclusion:**

there were no gender differences in the frequency of HTS visits. Social position for males and position in the family for females are suggested as the factors influencing HTS visits in Homa Bay County.

## Introduction

Human immunodeficiency virus (HIV) is a major global public health issue [1], that has taken over 35 million lives so far. Globally, approximately 37.7 million people were living with HIV by the end of 2020, with 1.5 million cases of newly infected reported during the year. Sub-Saharan Africa accounts for almost two-thirds of new HIV infections globally [1]. It was expected that by 2020, at least 90% of people living with HIV know their HIV status. This would have been possible if people were tested for their HIV infection status [2]. In 2019, only 75% of all people living with HIV were aware of their infection morbidity status [3]. In Kenya however, more than half (53%) of the 1.6 million people living with HIV in Kenya in 2019 were not aware of their HIV status. Yet, Kenya is one of the countries with the highest rates of HIV infection in the world, with an estimated adult prevalence of 4.9% [4].

**HIV testing services:** the Centres for Disease Control and Prevention (CDC) recommends that everyone aged between 13 to 64 years be tested for HIV at least once as part of routine health care. For those with specific risk factors, the CDC recommends testing at least once a year [5]. Annual HIV testing in high-prevalence settings is recommended for individuals at continued risk of infection to ensure early detection and initiation of antiretroviral therapy (ART) [6]. The HIV testing and treatment coverage is however lower among men than in women a trend that jeopardizes the health and lives of both men and their partners [7].

In Kenya, the National Acquired Immunodeficiency Syndrome (AIDS) and Sexually Transmitted Infections Control Programme (NASCOP) recommends that the general population visit the HIV testing service (HTS) centre at least once a year [8]. The term HTS is used to indicate the full range of services offered alongside HIV testing. This includes counselling (pre- and post-testing), linkage to appropriate HIV prevention, care and treatment services and other clinical support services, and coordination with laboratory services to support quality assurance and delivery of correct results [8].

**Barriers and facilitators of visits to HIV testing services: gender differences:** the barriers or facilitators of HTS visits in one population group are not necessarily the same in others. Health behaviour is influenced by multiple individual-level factors, such as gender, inter-individual factors, and community aspects. Different groups have different values and cultures; thus, it is necessary to examine related factors [9]. Gender is one of the most important factors regarding HTS visits. In Africa, the HIV testing rates of females have significantly improved as epidemiological and social problems have been identified [10]. Thus, many HIV prevention measures for females, such as maternal HIV testing, have been undertaken [11]. The testing rate could be improved by developing a program that considers gender differences. These differences, however, have yet to be studied in-depth.

While several studies have addressed facilitators and barriers affecting HTS visits by gender, some have targeted special groups, such as men who have sex with men [12,13], drug users [14], and sex workers [12,15-24]. Few have compared males and females in terms of facilitators and barriers [25-27]. Most previous studies have targeted both males and females but have not analyzed the data separately to consider gender differences [28-34]. Others targeted only males [35,36] or only females [37]. In addition, no study has investigated factors affecting visits among the general Kenyan population. Yet, Kenya is one of the countries with the highest HIV infection rates in sub-Saharan Africa.

This study is the first to investigate the frequency of HTS visits and its affecting factors among the general Kenyan population while also considering gender differences. According to the HTS national guideline in Kenya, each county should identify their target population, communication needs, and design of appropriate messages for the respective target groups [8]. Since the prevalence of HIV discordance couples is high in Kenya [38], they are one of the target populations. Although the effect of couple testing has been reported [39], it is possible that some individuals have been overlooked. Unlike men, women routinely receive HTS during their antenatal care and postnatal visits. In Homa Bay County, which has an established system for couple testing, antenatal care, and postnatal care, it is necessary to consider other measures of promoting HTS among men to ensure that they are not overlooked in HIV testing. Therefore, it is essential to investigate other factors that may be contributing to the differences in the frequency of visits to HTS centres between men and women in Homa Bay County. A comprehensive understanding of these differences is necessary to inform gender-sensitive programmes promoting HTS visits among the general population.

**Study objective:** this study aimed at determining frequency of HTS visits and its related factors to HTS visits among adults in Homa Bay, Kenya.

## Methods

**Study design:** the study was a cross-sectional study design involving a quantitative survey phase (N=660) followed by a qualitative phase with a sub-sample of the survey respondents (N=20) among adults who visited HTS centres. The aim of the questionnaire was to assess the frequency of HTS visits and quantitatively determine factors associated with HTS visits by gender. The outcome variable was “frequency of HTS visits” measured as “at least once a year” or “once after several years”, with the later considered as the baseline. The semi-structured interviews were conducted to assess the reasons for and awareness of HTS visits and to qualitatively determine the factors associated with HTS visits by gender. Combining methods offer pragmatic advantages when exploring a complex problem [40].

**Study settings:** the study was conducted in Homa Bay County, Kenya between May and August of 2019. This county was chosen because it has the highest HIV infection rate in Kenya (25.7%) [8], about 4.5 times higher than that across Kenya. It is estimated that 10.4% of HIV-infected people in Kenya live in Homa Bay County [8].

### Quantitative study

**Study population description:** the study population included adults who visited HTS centres in Homa Bay County, selected through multi-stage sampling. In stages 1 and 2, sub-counties and facilities were sampled, respectively. In stage 3, the participants were selected.

**Stage 1: sampling of sub-counties:** Homa Bay Town, as well as the sub-counties of Ndhiwa and Rachuonyo North, were purposively sampled for the study. Homa Bay Town was selected because it has the highest population density. While Ndhiwa and Rachuonyo North have low population densities, they are the most populous sub-counties.

**Stage 2: sampling of facility:** one public hospital with the highest attendance of outpatients was purposively selected from each sub-county. These were A hospital in Homa Bay Town, B hospital in Ndhiwa sub-county, and C hospital in Rachuonyo North sub-county.

**Stage 3: selection of participants:** all adults aged 18 years and older who visited the HTS centres during the study period were selected to participate. Pregnant women at antenatal care were excluded.

**Sample size:** the Kenya demographic health survey reported that proportions for adequate HTS visits were 58.9% and 70.9% for males and females, respectively, in Homa Bay County [41]. Based on these data, the sample size required for this research was calculated as follows: 10 (independent variables) x 10 (participants required for one independent variable) ÷ 0.3 (proportion who have not been able to receive HTS within the past year) x 2 (males and females separately) = 600 (participants). Expecting a 10% non-response rate [42], the minimum sample size was set as 660 participants (330 males, 330 females).

**Data collection procedures:** a structured interview questionnaire, written in English and translated to the local language (Dholuo), was used to collect quantitative data. The survey lasted about 10-15 minutes. The questionnaire was originally developed based on the literature review [43]. Participants were asked how often they visited the HTS centre. The questionnaire also addressed potential barriers and facilitators in four domains, as suggested by Qiao *et al*. [44]: a) individual characteristics of age, religion, occupation, educational level, marital status, last sexual intercourse, first sexual intercourse, condom use, number of sexual partners, HTS awareness, awareness of HIV status, latest testing place, and affordability of HTS visit costs; b) family and partner (cohabitation with partner, talking about HIV with partner, partner´s attitude, couple testing, and family support); c) socialisation (being accompanied by a friend to HTS, peer models, and disclosure of HIV status to others); d) health system (time taken to get to an HTS centre and ease in visiting HTS centre).

**Pre-test and training of research assistants:** the pre-test was conducted with ten participants not included in the main study to confirm the face validity and feasibility of the questionnaire. The research assistants were HTS counselling local certificate holders from each of the study facilities.

**Data analysis:** descriptive statistics were calculated for all variables. The outcome variable was “frequency of HTS visits” measured as “at least once a year” or “once after several years”, with the later considered as the baseline. After confirming a lack of multicollinearity among the variables, a backward stepwise logistic regression analysis was conducted to determine gender differences and related predictor factors in HTS visits among adults. Age and facility were controlled for in the analysis model. All the predictor variables in the model were religion, occupation, education, marital status, number of sexual partners, age of first sexual intercourse, last sexual intercourse, use of condom, HTS awareness, HIV status, last testing place, living with partner, talking with partner, attitude of partner, couple testing, family support, friend accompaniment, peer models, disclosure, time to HTS, affordability of HTS visits, and ease of visiting HTS. These were selected through a backward stepwise logistic regression with a variable reduction method (p<0.10). The final model for males had 5 variables: being 'Protestant´, 'partner´s positive attitude to HTS´, 'having a friend to accompany participants for testing´, 'distance to HTS less than 30 minutes´, and 'distance to HTS less than 30 to 60 minutes´. While for females were 5 valuables: 'two to five months since last sexual intercourse´, 'being in a polygamous marriage´, 'not having married´, 'the last HIV test was the community HIV test´, and 'affordability of transport cost to HTS centre´. All statistical tests were two-sided with a 5% level of significance. Statistical analysis was performed using SPSS Statistics Version 23.0 for Windows. We excluded data from the participants who did not answer the essential items such as gender, age, and HTS visits status.

### Qualitative study

**Study population description:** the study population consisted of adults who responded to the survey in the quantitative study. The inclusion and exclusion criteria were the same as those in the quantitative study.

**Sample size:** initially, 20 adults were expected to participate in the qualitative interviews: 5 males and 5 females who had completed the questionnaire and were visiting HTS at least once a year, and 5 males and 5 females who were not visiting HTS at least once a year.

**Data collection procedures:** three research assistants interviewed participants in a private room where they felt comfortable answering questions. The interviews were primarily conducted in English, but also in the local language when required. Initially, each assistant selected a participant based on the inclusion and exclusion criteria and obtained their consent for the interview. A semi-structured interview guide was used; responses were recorded with an audio recorder. The overall time for each interview was about 30 minutes. Data were collected using an open-ended in-depth interview guide, originally developed by the four researchers based on the quantitative study. The interview guide consisted of broad questions: a) reason for HTS visit; b) awareness of the need for HTS; c) awareness of the frequency of HTS; d) facilitators of HTS visits; and e) barriers to HTS visits.

**Pre-test and training of research assistants:** the pre-test was conducted with two participants not included in the main study to guarantee uniformity of the interview method and procedure. The researcher attended the first one or two interviews, after which the assistant conducted them alone. The research assistants conducting interviews held HTS counselling local certificates from each of the study facilities.

**Data analysis:** qualitative data were thematically analysed using a content analysis method. All participants´ interviews were audio-taped and transcribed. In the analysis, the researcher extracted statements related to the study topics, such as reasons, awareness, and factors for HTS visits, from the transcripts. The researcher conceptualized these data into subcategories based on similarities and differences under the supervision of an experienced qualitative researcher. Finally, the subcategories were classified into four major categories, as suggested by Qiao *et al*. [44]. A quasi-statistical analysis was performed to count the number of times each item appeared regarding the participant characteristics. Representative, verbatim quotes were selected to illustrate key findings.

**Ethical considerations/declarations:** this research was approved by the Medical Research Ethics Review Board of Yokohama City University in Japan (reference number: A181100005). Further approval was granted at Kenyatta National Hospital-University of the Nairobi Ethics Research Committee (reference number: KNH-ERC/A/147). Approval was also obtained from The National Commission for Science, Technology and Innovation (reference number: NACOSTI/P/19/67020/29982) in Kenya. The study was conducted in accordance with the World Medical Association Helsinki Declaration and complied fully with ethical considerations regarding the study participants. Written and informed consent were obtained from study participants prior to their participation.

## Results

### Quantitative data

**Characteristics of study participants:** Table 1 shows characteristics of the participants. In total, 663 adult males and females met the inclusion criteria and were recruited for the study. Of these, 657 adults agreed to participate, and 645 (284 males, 361 females) completed the questionnaire (valid response rate: 97.3%). Of these, 269 (41.7%) were adults in their 20s, followed by 176 (27.3%) in their 30s. Regarding religion, 384 (59.5%) were affiliated with African indigenous churches. In terms of occupation, 219 (34.0%) were in small-scale businesses, followed by 120 farmers (18.6%). Furthermore, 316 (49.0%) had completed secondary school. Most participants (29; 46.0%) were in monogamous marriages.

**Table 1:** characteristics of participants in the quantitative study

	Total (n=645)		Male (n=284)		Female (n=361)	
	n	%	n	%	n	%
**Age**						
Below 20	70	10.9	30	10.6	40	11.1
20-29	269	41.7	97	34.2	172	47.6
30-39	176	27.3	88	31.0	88	24.4
40-49	98	15.2	48	16.9	50	13.9
50 and above	32	5.0	21	7.4	11	3.0
**Religion**						
Local Christian	384	59.5	162	57.0	222	61.5
Catholic	145	22.5	70	24.6	75	20.8
Protestant	59	9.1	32	11.3	27	7.5
Muslim	30	4.7	9	3.2	21	5.8
Others	24	3.7	10	3.5	14	3.9
Not available	3	0.5	1	0.4	2	0.6
**Occupation**						
Small-scale business	219	34.0	94	33.1	125	34.6
Farmer	120	18.6	58	20.4	62	17.2
Formally employed	89	13.8	47	16.5	42	11.6
Fishing industry	59	9.1	33	11.6	26	7.2
Housewife	57	8.8	0	0.0	57	15.8
Others	100	15.5	52	18.3	48	13.3
Not available	1	0.2	0	0.0	1	0.3
**Education**						
Tertiary +	98	15.2	55	19.4	43	11.9
Secondary education	316	49.0	142	50.0	174	48.2
Primary education	202	31.3	71	25.0	131	36.3
No formal education	27	4.2	14	4.9	13	3.6
Not available	2	0.3	2	0.7	0	0.0
**Marital status**						
Not married	226	35.0	101	35.6	125	34.6
Married (monogamous)	297	46.0	123	43.3	174	48.2
Married (polygamous)	115	17.8	56	19.7	59	16.3
Not available	7	1.1	4	1.4	3	0.8
**Health facility**						
Homa Bay Town	272	42.2	125	44.0	147	40.7
Ndhiwa	189	29.3	90	31.7	99	27.4
Rachuonyo North	184	28.5	69	24.3	115	31.9

**Figure 1:**
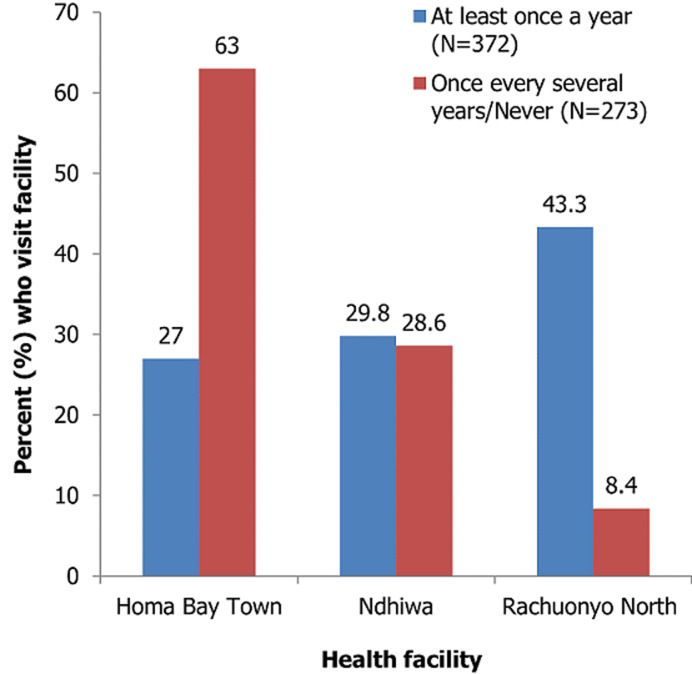
frequency of HTS visits by facility

**Frequency of HTS visits:** out of the 663 study participants, 372 (57.7%) visited the HTS centres at least once every year. The frequency of visits did not differ significantly by gender (56.3%, 58.7%, P=0.785). The frequency of HTS visits was however significantly higher at rural-based facilities (Rachuonyo North: 87.5%; Ndhiwa: 58.7%) compared to the urban-based facility (Homa Bay Town: 36.8%; P<0.001), as seen in Figure 1.

**Factors related to HTS visits:** study findings related to HTS visits are shown in Table 2. The logistic regression analysis revealed that for all adults in this study, the facilitators were 'within 2 to 5 months from last sexual intercourse´ (OR=2.24, 95% CI: 1.09-4.62, P=0.029), 'more than 6 months from last sexual intercourse´ (OR=4.69, 95% CI: 1.59-13.89, P=0.005) and 'accompaniment by a friend for testing´ (OR=2.07, 95% CI: 1.31-3.25, P=0.002). The barriers were 'fishing occupation´ (OR=0.36, 95% CI: 0.15-0.81, P=0.014), 'unmarried´ (OR=0.42, 95% CI: 0.24-0.73, P=0.002), 'no sexual partner´ (OR=0.15, 95% CI: 0.03-0.71, P=0.017), 'first sexual intercourse at age 20 to 24´ (OR=0.43, 95% CI: 0.22-0.86, P=0.016), 'unaware of HTS´ (OR=0.42, 95% CI: 0.20-0.89, P=0.022), 'last HIV test was the community HIV test´ (OR=0.37, 95% CI: 0.22-0.63, P<0.001), and 'affordability of transportation to HTS centre´ (OR=0.46, 95% CI: 0.26-0.80, P=0.006). For males, the facilitators were 'Protestant religion´ (OR=6.68, 95% CI: 1.56-28.54, P=0.010), 'partner´s positive attitude to HTS´ (OR=2.54, 95% CI: 1.07-5.99, P=0.034), and 'accompaniment by a friend for testing´ (OR=3.57, 95% CI: 1.46-8.72, P=0.005). The barriers were 'distance to HTS less than 30 minutes´ (OR=0.32, 95% CI: 0.11-0.95, P=0.040) and 'distance to HTS less than 30 to 60 minutes´ (OR=0.29, 95% CI: 0.11-0.73, P=0.009). For females, the facilitator was 'within 2 to 5 months from last sexual intercourse´ (OR=3.52, 95% CI: 1.20-10.36, P=0.022). The barriers were 'polygamous marriage´ (OR=0.26, 95% CI: 0.11-0.65, P=0.004), 'unmarried´ (OR=0.35, 95% CI: 0.16-0.75, P=0.007), 'last HIV test was the community HIV test´ (OR=0.20, 95% CI: 0.09-0.45, P<0.001), and 'affordability of transportation to HTS centre´ (OR=0.29, 95% CI: 0.12-0.70, P=0.006).

**Table 2:** factors related to HTS visits

		Total (n=645)				Male (n=284)				Female (n=361)			
		95% CI				95% CI				95% CI			
Factors		OR	Lower	Upper	P-value	OR	Lower	Upper	P-value	OR	Lower	Upper	P-value
**Individual**													
Religion (Catholic)	Protestant	-	-	-	-	6.68	1.56	28.54	0.010	-	-	-	-
	Local Christian	-	-	-	-	1.06	0.48	2.37	0.884	-	-	-	-
	Muslim	-	-	-	-	0.95	0.10	9.30	0.963	-	-	-	-
	Others	-	-	-	-	0.17	0.02	1.25	0.082	-	-	-	-
Occupation (farmer)	Fishing industry	0.35	0.15	0.81	0.014	0.46	0.13	1.65	0.231	-	-	-	-
	Small-scale business	0.85	0.48	1.53	0.594	0.99	0.38	2.63	0.988	-	-	-	-
	Formally employed	1.46	0.69	3.09	0.323	3.10	0.97	9.91	0.056	-	-	-	-
	Others	1.25	0.58	2.71	0.566	5.04	0.92	27.60	0.062	-	-	-	-
Marital status (monogamous)	Married (polygamous)	0.60	0.33	1.07	0.085	-	-	-	-	0.26	0.11	0.65	0.004
	Not married †	0.42	0.24	0.73	0.002	-	-	-	-	0.35	0.16	0.75	0.007
Number of sexual partner (one)	None	0.15	0.03	0.71	0.017	-	-	-	-	-	-	-	-
	Two or more	0.73	0.47	1.15	0.174	-	-	-	-	-	-	-	-
Age of first sexual intercourse (15-19 years old)	Younger than 14 years old	0.80	0.49	1.29	0.352	-	-	-	-	-	-	-	-
	20-24 years old	0.36	0.18	0.76	0.007	-	-	-	-	-	-	-	-
	Older than 25 years old	0.65	0.22	1.94	0.439	-	-	-	-	-	-	-	-
Last sexual intercourse (within 1 week)	Within 1 month	1.17	0.73	1.88	0.509	-	-	-	-	1.52	0.72	3.20	0.276
	Within 2-5 months	2.24	1.09	4.62	0.029	-	-	-	-	3.52	1.20	10.36	0.022
	More than 6 months	4.69	1.59	13.89	0.005	-	-	-	-	2.58	0.71	9.39	0.151
HTS awareness (yes)	No	0.42	0.20	0.88	0.022	-	-	-	-	0.27	0.07	1.09	0.066
HIV status (known)	Not known	-	-	-	-	3.57	0.81	15.71	0.092	-	-	-	-
Latest testing place (public hospital)	Private hospital	1.10	1.10	0.56	0.785	-	-	-	-	0.92	0.34	2.51	0.874
	In the community	0.37	0.22	0.63	0.000	-	-	-	-	0.20	0.09	0.45	<0.001
	Home	0.76	0.35	1.65	0.484	-	-	-	-	1.16	0.34	3.93	0.813
Affordability of cost of HTS visits (yes)	No	0.46	0.26	0.80	0.006	-	-	-	-	0.29	0.12	0.70	0.006
**Family and partner**													
Attitude of partner (negative)	Positive	-	-	-	-	2.54	1.07	5.99	0.034	-	-	-	-
**Socialisation**													
Friends to accompany to HTS (without)	With	2.07	1.31	3.25	0.002	3.57	1.46	8.72	0.005	-	-	-	-
Peer model (without)	With	-	-	-	-	2.11	0.99	4.52	0.054	-	-	-	-
**Health system**													
Time to HTS (> 60 minutes)	30 minutes >	-	-	-	-	0.32	0.11	0.95	0.040	-	-	-	-
	30-60 minutes	-	-	-	-	0.29	0.11	0.73	0.009	-	-	-	-

OR=odds ratio; CI=confidence interval; HTS=HIV testing services; † not married includes single or divorced or widowed; input variables: religion, occupation, education, marital status, no. of sexual partner, age of first sexual intercourse, last sexual intercourse, use of condom, HTS awareness, HIV status, last testing place, living with partner, talking with partner, attitude of partner, couple testing, family support, friends to accompany to HTS, peer model, disclosure, time to HTS, affordability of cost of HTS visits, easy to HTS; age and facility were controlled simultaneously for potential confounders; Hosmer-Lemeshow test of goodness of fit; total: P=0.047; male: P=0.924; female: P=0.027

### Qualitative data

**Characteristics of study participants:** the qualitative study included 17 participants (8 males, 9 females). The average age was 33 years (20-54). Each interview lasted about 13.6 (8-21) minutes. The most frequently stated reason for visiting an HTS centre was 'to know my status´ (13/17 participants). Two male participants indicated the reason as 'for a check-up´, and another two reported that it was 'to see what the HTS is´. Regarding the importance of visiting an HTS centre, those surveyed considered it 'very important´ (3/17), 'important´ (11/17), and 'not important´ (3/17).

**Gender differences in factors influencing HTS visits:** for males and females, the most frequently mentioned factor was 'affordability of transportation to the HTS´ (Table 3). For males, the second most frequent factor was 'fear of stigmatization´, followed by 'fear of receiving the test results´. However, for females, the second most frequent factor was 'other commitments´. In the category level, individual factors were most frequently mentioned by both males and females, followed by socialisation factors for males and the health system for females.

**Table 3:** factors affecting HTS visits reported in qualitative interviews

Factors	Male (n=8)	Female (n=9)
**Individual**		
Affordability of transport to go to the HTS	8	9
Fear of receiving HIV testing results	5	5
Other commitments	4	6
Willingness to know status	3	2
Knowledge of importance of HTS	2	3
Feeling sick/injury	2	3
Attitude among adults and males	2	2
Unwillingness to disclose HIV status to others	2	-
Financial constraints for food and transport	1	4
Love their body and life	1	2
See their body as healthy	1	-
Forgot the appointment date	-	2
If previous result was HIV negative	-	1
**Partner and family**		
Worried about children's future	2	3
Fear of sharing results with partner	2	1
Family responsibility	1	-
**Socialisation**		
Fear of stigmatisation	6	2
Availability of someone to talk to about HIV	4	-
Traditions and cultural beliefs	3	-
Motivation from community, media, church, or leaders	2	2
Memories of witnessing death from HIV/AIDS	1	2
**Health system**		
Distance to HTS centre	4	4
Counselling	3	2
Confidentiality	2	3
Attitude of health service providers	2	2
Need for “door-to-door testing”	1	3
Awareness meetings	-	1
Fear of long queues for ARVs	-	1
Non-functional facilities	-	1

HTS: HIV testing services; ARVs: anti-retroviral drugs

Below, participants´ quotes demonstrate the factors that differed by gender in terms of frequency. Regarding 'fear of stigmatization´, a man in his 40s mentioned the following: *”you find someone afraid to go there (HTS centre) because they think people will know their status. Other people who know them (at the HTS centre) may see them. They think these people can make their (visitor´s) status known to the community”*.

The same man in his 40s discussed 'having significant others to talk to about HIV´, a factor in the socialisation category: *“the advantage of friends is that sometimes your friend can advise you to go to the hospital (for HIV testing)”*.

Another male participant in his 20s commented on 'tradition and cultural belief´, also in the socialisation category: *”older people used to say that it was Chira (taboo), but they have come to realize that this thing is not a taboo. It is HIV, which is killing people”*.

A female participant in her 30s mentioned 'financial ability´ in the individual category and 'concerns for children´s future´ in the partner and family categories: *”we take the money to buy food for the children. We should not use the money for testing when the children cannot eat. Such things make that people do not come for testing”*.

In the health system category, 'need for door-to-door testing´ and 'sensitisation forums´ were discussed by a woman in her 20s: *”(it will motivate us to have HIV testing) when you bring to the community (outreach) door-to-door testing, provide free gifts like medication, or call them for seminars or workshops”*.

## Discussion

The quantitative and qualitative analyses revealed similar concepts: regarding HTS visits, males are concerned about social position, whereas females are more concerned about their economic strength and position within their family. For males, these findings show that protecting one´s social position is a facilitator, while anticipated threat is a barrier. According to the Kenya National Bureau of Statistics [41], 26.1% of residents accept people living with HIV/AIDS, suggesting that stigma, prejudice, and discrimination still exist in Kenya. Camlin *et al*. [36] discussed HIV risks, with many men questioning 'traditional´ masculine gender norms that enhanced risks. Barnabas *et al*. [28] qualitatively determined that masculinity is a barrier to HTS visits. In other words, this study revealed that existing gender norms, feelings of fear, and the culture in Homa Bay County influence male involvement in HTS.

Peers with whom an individual worked or interacted with regularly easily recognized symptoms, recommended testing, and sometimes offered to 'escort´ participants to the test [35]. The concept of 'homosociality´ describes social bonds between same-sex persons. Horizontal 'homosociality´ points towards more inclusive relations between males based on emotional closeness, intimacy, and a non-profitable friendship [45]. This concept could be useful regarding males´ involvement. Clearly, being accepted by society has a great influence on men´s pride. This finding suggests 'peer networking´ or 'friends testing´ projects involving the joining of peers or friends as potential approaches to HIV testing and HTS visits.

For females, weak position within the family is a barrier. This involves competing needs, such as provision of food for the family, which was defined by Falnes *et al*. [46] as a mother´s responsibility. Females need to work and save money for children´s food and must balance many things within the family, such as finances and time, which then impact HTS visits. However, females with high positions within their families can easily express their desires. In previous studies by Agha [37] and Tabana *et al*. [25], higher education, financial wealth, and urban living were the main facilitators.

For those with difficulties visiting HTS, such as low familial positions and less economic strength, community outreach programmes have been developed. However, the sustainability of community outreach is likely unrealistic due to high project management costs. Furthermore, Dellar *et al*. [47] argued that the evaluation designs of these interventions are generally weak, and no evidence confirms their effectiveness. Accordingly, people who have been tested in the community should be encouraged to visit HTS facilities. It is imperative that community outreach initiatives refer people to HTS centres for future visits.

The fact that more than half of all participants who were not likely to visit the HTS centre once every year were from Homa Bay Town was an unexpected finding. The urban population was expected to be more aware of the benefits of regular HTS visits compared to Rachuonyo North. However, the high prevalence of HIV/AIDS in Rachuonyo North sub-county is accompanied by a heavy presence of health agencies implementing HIV/AIDS-related activities, including sensitisation on the need for HTS visits.

We found that many people do not receive proper HIV testing. Thus, a programme focused on increasing HTS visits is crucial. The current results may reflect the impact of existing measures on promoting HTS visits in rural areas. Currently, a renewed focus on HIV and implementation of HIV-related interventions in urban areas is necessary. There were no studies investigating specific factors affecting HTS visits among adults in Kenya until now. Thus, this study can guide gender-sensitive programming. Compared with a review article that analysed the factors behind HIV testing behaviour in Africa [43], this study did not show age or family responsibilities as factors among males; however, men´s senses of masculinity and women´s economic burdens were common. In particular, regarding the abstracted factor of masculinity, this study suggested the importance of friends and peers as a concrete approach method.

**Limitations:** readers should be aware of some limitations of this study. First, the study focused only on the frequency of HTS visits by individuals who came for HIV testing, not on the HIV testing rate. In this respect, we recommend focusing on people who have never been tested for HIV to determine barriers and facilitators and develop an intervention method. In addition, while stigmatization and fear were identified as factors affecting HTS visits in both the quantitative and qualitative studies, we could not directly ask about stigma in the questionnaire because there was no suitable measurement for it. Further research is necessary to determine the severity of fear and stigmatization.

## Conclusion

In Homa Bay, Kenya, slightly more than half of the study population visited HTS centres at least once a year as per the NASCOP recommendations. There was no significant difference between the frequency of visits between males and females. However, there were gender differences between the factors associated with HTS visits. Social position (for males), as well as economic strength and position in the family (for females), are suggested as factors. This study provides results that can guide gender-sensitive programs for HTS visits.

### What is known about this topic

- Kenya has one of the highest rates of HIV infection in the world, with an estimated adult prevalence of 5.9%;
- Homa Bay County has the highest HIV infection rate in Kenya, at 25.7%;
- Previous studies identified facilitators and barriers of HTS visits among special groups such as men who have sex with men, drug users, and sex workers in Africa.

### What this study adds

- While previous studies have shown that there are gender differences in HTS visits, this study shows that such differences are not there;
- This study demonstrates that there are differences in factors associated with HTS visits by gender;
- The HIV prevention and management programs should aim at understanding and addressing the aforesaid factors in the context of rural and urban settings, separately, rather than narrowly focusing on improving HTS visits by gender.
